# 肺癌细胞中HGF/c-Met信号通路的异常调控及其靶向药物研究进展

**DOI:** 10.3779/j.issn.1009-3419.2014.08.08

**Published:** 2014-08-20

**Affiliations:** 300052 天津, 天津医科大学总医院, 天津市肺癌研究所, 天津市肺癌转移与肿瘤微环境实验室 Tianjin Key Laboratory of Lung Cancer Metastasis and Tumor Microenvironment, Tianjin Lung Cancer Institute, Tianjin Medical University General Hospital, Tianjin 300052, China

**Keywords:** c-Met, HGF, 靶向药物, 肺肿瘤, c-Met, HGF, Targeting Drugs, Lung neoplasms

## Abstract

c-MET是原癌基因c-MET编码的蛋白产物, 是肝细胞生长因子(hepatocyte growth factor, HGF)受体, 具有络氨酸激酶活性。c-Met的异常表达与肺癌的发生发展有着密切的关系。HGF与其c-Met受体结合后, 活化c-Met酪氨酸激酶活性, 能促进多种肿瘤细胞包括肺癌细胞的增殖、新生血管生成及肿瘤侵袭和迁移。针对HGF/c-Met信号转导通路的靶向治疗是目前肺癌治疗的新热点。本文将就HGF/c-Met信号转导通路在肺癌中异常调控及其靶向药物在肺癌中的研究进展进行综述。

## Abstract (en)

C-MET is a coding product of proto oncogene c-MET, hepatocyte growth factor (HGF) receptor with tyrosine kinase activity.The abnormal expression of *c-Met* gene is correlated with the tumorigenesis and development of lung cancer.Once the tyrosine kinase is activated by the interaction between the HGF ligand and the TK receptor, and the activated kinase will promote the cell proliferation, angiogenesis, invasion and metastasis of different tumors, as well as lung cancer. The targeted therapy to HGF/c-Met signal pathway is a new highlight in the treatments of lung cancer.In this review, we will discuss the dysregulation of HGF/c-Met signal pathway in lung cancer and the new progress for the targeted drugs to this pathway.

肺癌是全球范围内发病率和死亡率最高的恶性肿瘤之一, 尽管外科手术技术不断提高, 化疗新药不断上市并进入临床应用, 但肺癌患者的预后仍然很差, 原因是肺癌的发病机理复杂, 涉及多种信号通路及分子的异常调控。肝细胞生长因子(hepatocyte growth factor, HGF)是一种多肽生长因子, 具有强促分裂、诱导上皮细胞迁移、侵袭以及诱导血管生成等作用, 其生物学活性由其受体c-Met所介导。原癌基因*c-Met*属于具酪氨酸激酶活性的生长因子受体。HGF/c-Met信号通路在多种肿瘤包括肺癌组织中出现异常活化, 涉及*c-Met*基因的过表达、扩增及突变等, 通过一系列的信号转导, 促进肿瘤的生长、侵袭和转移, 阻断HGF/c-Met信号途径可有效抑制肿瘤的发生发展和转移。

本文就HGF/C-Met信号通路的异常活化与肺癌发生发展的关系及其靶向药物在肿瘤治疗中的研究进展进行综述。

## 1 HGF/c-Met的结构和生理功能

HGF属于可溶性细胞因子家族, 是纤溶酶原相关生长因子家族成员之一, 1989年由日本学者从大鼠血浆中提取并成功克隆, 又名扩散因子(scatter factor, SF)^[1, 2]^。人HGF基因位于7号染色体(7q21.1)上, 由18个外显子和17个内含子构成, 长度约为70 kDa, 编码728个氨基酸的糖蛋白。HGF由分子量为69 kDa的α链和分子量为34 kDa的β链通过二硫键连接成异二聚体^[3]^。HGF包含6个结构域:1个N-末端发卡环结构域(HL)、4个Kringle结构域以及1个丝氨酸蛋白酶类似物结构域(serine protease analogue structure domain, SPH)(图 1a)^[4]^。其中α链有4个圈形区(kringle), N端有一发夹样结构, 该发夹样结构与前2个kringle区结构为HGF发挥生物学效应所必需的。β链是与c-Met结合的位点, 具有丝氨酸蛋白酶样结构, 与丝氨酸蛋白酶原的催化区具有高度的同源性但不具备蛋白酶的催化活性, 缺乏β链的HGF丧失应有的生物学作用。

**1:**
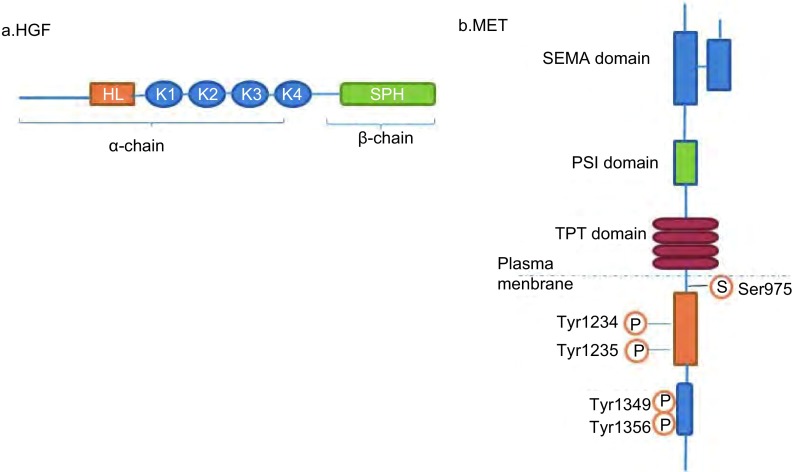
HGF(a)和c-Met(b)结构图 The structures of HGF (a) and c-Met (b).HGF:Hepatocyte growth factor.

c-Met是由原癌基因*c-Met*编码的蛋白产物, 具有酪氨酸激酶的活性^[5, 6]^, 被配体激活后介导细胞信息传递, 是细胞增殖、分化和运动的重要调节因素。在胚胎发育或组织器官发生损伤的情况下, 如肝脏、肾脏和肺组织损伤时, c-Met被诱导激活参与创伤愈合及组织修复过程^[7, 8]^。*c-Met*基因位于7号染色体(7q21-q31)上, 由21个外显子和20个内含子构成, 长度约为120 kDa。c-Met初级转录本生成150 kDa的多肽, 然后部分糖基化形成170 kDa的单链前体蛋白。该前体蛋白进一步糖基化形成由50 kDa的胞外链(α链)与145 kDa的跨膜链(β链)连结而成的约190 kDa的异二聚体^[9, 10]^。跨膜链(β链)包括SEMA结构域(sema homology region, SEMA)、PSI结构域(plexin-semaphorin-integrin, PSI)、4个免疫球蛋白样重复结构域(immunoglobulin-like regions in plexins and transcription factors, IPT)、一个跨膜域、一个近膜域(juxtamembrane domain, JM)、酪氨酸激酶结构域(tyrosine kinase, TK)和一个羧基末端的尾部区域(Carboxyl terminal, CT)(图 1b)。其中SEMA结构域是配体结合的重要元素之一, 被认为是HGF的结合位点, 而PSI结构域的功能是使c-Met的胞外片段很好地与配体结合。近膜域(JM)包含两个蛋白磷酸化位点:S985和Y1003。S985位点发生磷酸化可负调控激酶活性, Y1003残基磷酸化后与c-Cbl结合。c-Cbl是一种E3泛素连接酶, 可以通过指环结构域与E2泛素结合酶结合, 进而支配细胞内摄并最终导致底物泛素化和降解。酪氨酸激酶结构域活化位点Y1230、Y1234和Y1235的磷酸化促进酪氨酸激酶的激活。c-Met与HGF结合后, c-Met的活化残基Y1234和Y1235发生磷酸化, 进而招募胞内SH2结构域(Src homology-2, SH2)和其他特异的信号效应分子激活下游信号通路^[11]^。

HGF主要在间质细胞中表达, c-Met主要在各种上皮细胞中表达。HGF与c-Met受体特异性结合后诱导c-Met蛋白发生构象改变, 激活受体胞内蛋白激酶结构域中的酪氨酸蛋白激酶(protein tyrosine kinase, PTK), 从而暴露下游信号分子的多功能对接位点(multisubstrate docking site, MDS), 磷脂酰肌醇激酶(phosphatidyl inositol 3-kinase, PI3K)、生长因子受体结合蛋白-1(Grb2-associated binder 1, Gab1)、生长因子受体结合蛋白-2(Grb2-associated binder 2, Gab2)等在MDS聚集并结合, 进而激活Gab2-Ras和Gab1-PI3K等信号转导通路^[12]^。c-Met受体的激活, 信号经级联式磷酸化反应, 将信号逐级放大, 最终转入细胞核内的转录机构, 调节细胞的增殖、分化、收缩、运动、分泌及分裂等多种生物学行为。

HGF/c-Met在胚胎发育、器官形态以及血管发生等生理过程中发挥重要作用。Schmidt等^[13]^研究发现, HGF的突变可使小鼠胎盘发育尤其是滋养层细胞的发育受阻, 从而影响小鼠胚胎发育, 最终导致小鼠胎盘及肝脏严重受损, 小鼠在胚胎期即死亡。可见, HGF在胚胎发育过程中有着重要的功能。HGF还与器官发育有关, HGF通过调节上皮细胞与间充质细胞之间的相互作用而调节器官发育过程。在发育过程中, c-Met在多种器官上皮细胞中表达, HGF由临近的间充质细胞分泌。Yang等^[14]^发现HGF能促使乳腺导管分支的形成, 抑制分泌蛋白的产生。阻断内生性HGF的表达, 可阻碍乳腺导管分支结构的形成。HGF/c-Met系统还可促进新生血管的形成, Bussolino等^[15]^研究发现, 体内HGF缺失可导致止血血栓、炎症等, 体内HGF可诱导兔角膜新生血管的形成。

## 2 c-Met在肺癌组织中的异常调控

c-Met在癌细胞中的调控机制不同于正常细胞, 研究发现c-Met介导异常信号转导在多种肿瘤包括肺癌中起着重要作用, HGF依赖的c-Met信号通路的活化可以激活下游通路, 如丝氨酸/苏氨酸蛋白激酶(serine/threonine-specific protein kinase, AKT)、胞外信号激酶(extracellular signal-regulated kinase, ERK)、PI3K/AKT、MAPK信号通路等, 从而介导肿瘤发生、侵袭和转移、血管新生、上皮-间质转化等过程^[16]^(图 2)。c-Met在肺癌细胞中的异常调控机制有多种, 主要有:c-Met过表达、*c-Met*基因扩增和*c-Met*基因突变等。

**2:**
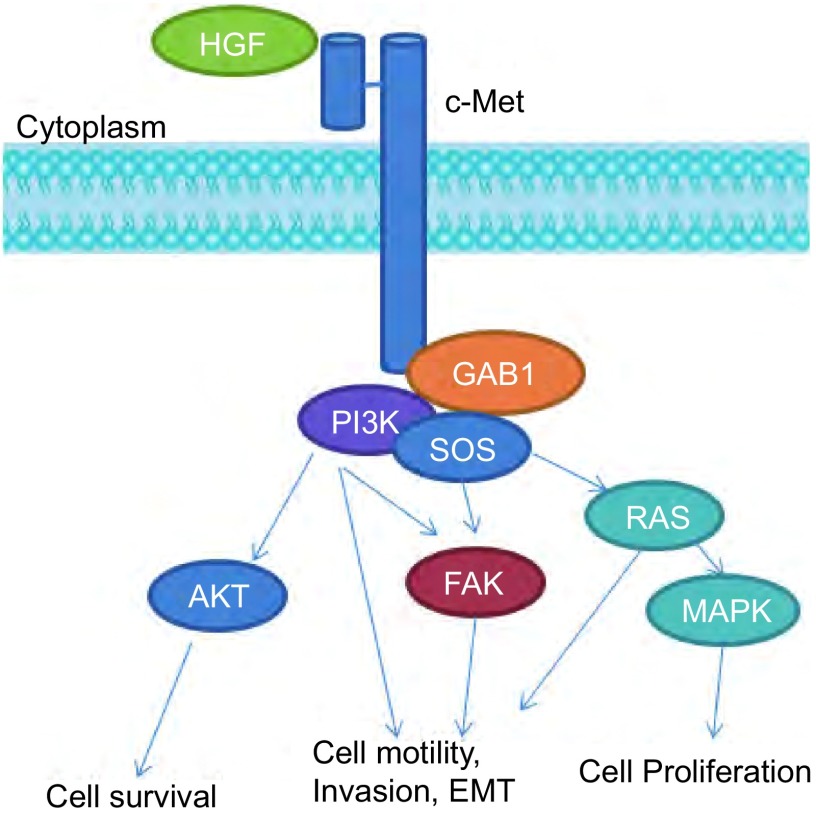
HGF/c-Met信号通路示意图 The HGF/c-Met signaling pathway.GAB1:Grb2-associated binder 1;PI3K:phosphatidylinositol 3-hydroxy kinase; SOS:Son of Sevenless; Akt:serine-threonine protein kinase; FAK:Focal Adhesion Kinase; RAS:ras gene family; MAPK:mitogen-activated protein kinases.

### 2.1 c-Met的过表达

c-Met的过表达现象在多种肿瘤包括肺癌中都存在。Ma等^[17, 18]^研究分析发现, c-MET在非小细胞肺癌组织中约61%呈高表达, 而在小细胞肺癌中约25%呈高表达。非小细胞肺癌中约67%腺癌、57%大细胞癌、57%鳞癌呈c-MET高表达。肺腺癌的其他研究^[19, 20]^也报道, 41-72%的肺癌患者存在c-MET表达, 而25-67%患者c-MET呈过表达现象。c-Met过表达可在体外导致原代成骨细胞发生骨肉瘤样细胞转化, 也可以导致骨肉瘤相似疾病的发生^[21]^。而在肺癌中, c-Met的过表达常常与肿瘤分期晚和预后不良有关。在非小细胞肺癌组织中, 与对应的癌旁组织相比, c-Met的表达水平高2倍-10倍, 而HGF的表达水平高10倍-100倍^[22]^。Park等^[23]^通过荧光免疫杂交方法和免疫组化等方法研究380例非小细胞肺癌患者组织样本, 发现13.7%的患者存在c-Met的高表达, 且这些患者的生存时间和无病生存期较短, 提示c-Met的扩增和过表达是非小细胞肺癌患者的不良预后因素。Nakamura等^[24]^研究了130例非小细胞肺癌患者组织样本中c-Met和磷酸化c-Met的表达情况, 发现HGF和c-Met高表达与乳头状结构相关, 与腺癌分化程度无明显相关性; 而磷酸化c-Met与腺癌的分化程度和乳头状结构均有相关性, 且磷酸化c-Met的表达水平与磷酸化AKT相关。多种因素可影响c-Met的表达, Pennacchietti等^[25]^指出肿瘤内的缺氧区域存在c-Met过表达现象, 低氧活化作用可诱导c-Met的转录, 从而引起HGF/c-Met信号通路的放大, 造成c-Met的过表达。抑制c-Met的表达, 低氧诱导的肿瘤侵袭生长将被抑制。PAX5是一种B细胞发育所需的核转录因子, 在小细胞肺癌中高表达。PAX5在很多肺癌组织中常常与c-Met或磷酸化c-Met共表达, 在中分化或中-高分化的神经内分泌肿瘤包括一些非典型性良性肿瘤、小细胞肺癌以及大细胞神经内分泌肿瘤中, PAX转录因子可调控c-Met的转录^[26]^。

另外, 循环系统中c-Met的高表达也与非小细胞肺癌存在密切关系。Cheng等^[27]^发现75%(34/45)的非小细胞肺癌患者肿瘤组织样本中存在c-Met过表达现象, 并且这34例患者中有68%(23/34)在循环系统中也能检测到c-Met的高表达。进一步分析发现循环系统中c-Met与淋巴结分期的关系, 提出循环系统c-Met过表达是肿瘤早期复发的重要因素。提示可以将循环系统中c-Met作为分子标志物用于肺癌特别是早期肺癌复发的筛查。多项研究^[28, 29]^均提示循环系统中c-Met与其他分子标志物的结合可作为肺癌早期筛查的手段。

### 2.2 c-Met的扩增

c-Met扩增也是c-Met异常调控的重要机制之一。研究表明, c-Met的扩增是非小细胞肺癌患者的预后不良因素之一。约22%的表皮生长因子酪氨酸激酶抑制剂(epidermal growth factor receptor-tyrosine kinase inhibitors, EGFR-TKIs)获得性耐药的非小细胞肺癌患者中存在c-Met扩增。c-Met扩增可引起吉非替尼耐药^[30]^。Pao等^[31]^提出, 吉非替尼使表皮生长因子受体(epidermal growth factor receptor, EGFR)信号通路阻滞, 肺癌细胞转而依赖c-Met信号通路活化, 以维持细胞的生长。c-Met的扩增导致c-Met受体的过表达, 并激活下游通路的信号转导, 特别是PI3K/AKT通路, 细胞出现获得性耐药现象。

### 2.3 c-Met突变

c-Met异常调控的另一个机制是*c-Met*突变, 在非小细胞肺癌中的出现比例约为5%-10%。c-Met的突变可发生在胞外区也可发生在胞浆区。c-Met胞外SEMA结构域是受体激活和二聚化所必须的, 发生在该结构域的突变有多种类型, 如N375S、M431V以及N454I等。有研究者^[32]^分析了141个东亚人、76个高加索人和66个非裔美国人肺癌患者中的肺癌组织基因组DNA, 发现N375S是一个出现频率很高的突变类型, 且东亚人比高加索人更容易出现N375S突变, 而在非裔美国人患者却未见有该突变类型。进一步研究发现, N375S在鳞癌中的突变率要远高于腺癌和大细胞癌, 且在鳞癌病例中, N375S突变在吸烟患者中发生率高。c-Met JM结构域的突变较常见于急性髓性白血病中^[33]^。Lee等^[34]^将含T992I突变的3T3细胞注射入无胸腺小鼠中, 与野生型3T3细胞比较, c-Met T992I突变能加速肿瘤的形成。Ma等^[18]^分析大细胞肺癌组织和细胞系中c-Met的突变情况, 发现c-Met JM结构域中存在T992I、R970C和S1010P突变, 且R970C突变只存在于非裔美国人和高加索人, 而不存在于亚洲人。

## 3 HGF/c-Met信号通路异常与肺癌发生、发展的关系

HGF/c-Met除了参与调节正常细胞粘附、分化和迁移外, 也参与细胞的恶性转化。HGF激活的c-Met受体与细胞内的一些靶蛋白直接作用, 可激活包括Ras/MAPK和PI3K/PKB等信号转导的各级联途径, 调节多种恶性肿瘤细胞的增殖分化、形态变化和运动侵袭等, 从而促进肿瘤的浸润与转移。多项研究表明, HGF/c-Met与多种肿瘤的发生、发展和转移密切相关。

### 3.1 HGF/c-Met诱导肿瘤的增殖和发生

原癌基因*c-Met*特异性结合HGF后, c-Met蛋白受到诱导而发生构象改变, 激活其胞内蛋白激酶结构域中的酪氨酸激酶PTK, 被激活的PTK先使受体自身的酪氨酸残基(Tyr)磷酸化, 从而激活下游信号通路如PI3K/AKT、Ras/MAPK等, 最后转导至细胞核内, 导致肿瘤细胞的增殖和分化。吸烟是公认的肺癌的危险因素之一。有研究^[35]^发现, 烟草中的尼古丁可以上调肺癌组织和肺泡Ⅱ型细胞中的HGF表达, 从而使HGF/c-Met信号通路发生异常改变, 导致肺癌的发生。Stabile等^[36]^将有HGF过度表达的转基因组小鼠与野生组小鼠相对比, 发现转基因小鼠组对致癌物质(烟草中的NNK)的易感性明显增高, 肺癌的发生率也提高。说明HGF/c-Met信号通路在肺癌形成过程中起明显促进作用。

### 3.2 HGF/c-Met诱导肿瘤新生血管的生成

新生血管的形成对原发肿瘤细胞的生成和增殖是必不可少的, 同时也是肿瘤侵袭转移的必要条件。HGF可以激活血管内皮细胞并引起血管内皮细胞的增殖和迁移, 从而参与肿瘤新生血管的生成, 或调节其他血管生长因子, 如血管内皮生长因子(vascular endothelial growth factor, VEGF)的表达水平, 间接促进肿瘤新生血管的生成。Bussolino等^[15]^的研究发现, HGF与内皮细胞表面的c-Met受体结合, 激活受体酪氨酸激酶并发生磷酸化, 直接诱导血管内皮细胞的增殖。同时发现HGF通过刺激内皮细胞的增殖及促进其迁移, 参与内皮细胞损伤修复机制。

### 3.3 HGF/c-Met诱导细胞外基质(extracellular matrix, ECM)的降解

细胞外基质为肿瘤转移的重要组织屏障, 降解细胞外基质是肿瘤细胞侵袭、转移的重要步骤。基质金属蛋白酶(matrix metallopotease, MMP)和纤溶酶原激活物(plasminogen activator, PA)是影响肿瘤转移的两种重要的蛋白水解酶。MMP的过度表达或活性提高与肿瘤生长、浸润和转移有密切关系。Kermorgant等^[37]^发现用HGF刺激人结肠癌上皮细胞可大大增加了细胞MMP1、MMP2、MMP9的表达量, 使细胞侵袭力增强。Reid等^[38]^研究发现HGF能够诱导细胞内尿激酶启动子活性及尿激酶的分泌, 从而促使细胞ECM的降解, 最终促进肿瘤的转移。

### 3.4 HGF/c-Met与肺癌侵袭转移的关系

异常活化的HGF/c-Met信号转导与肺癌发生、侵润和转移有着密切联系。Wang等^[39]^研究发现, c-Met在小细胞肺癌组织和细胞中高表达, 用siRNA技术敲除c-Met的表达, 发现肺癌细胞的增殖和侵袭能力下降, 从而证实c-Met与肺癌细胞侵袭能力的关系。Gumustekin等^[40]^发现c-Met的表达与肺癌的淋巴结侵袭以及ras同源基因家族成员A(ras homolog gene family member A, RhoA)和金属蛋白酶组织抑制剂-3(tissue inhibitor of metalloproteinase-3, TIMP-3)表达相关, 提示HGF/c-Met信号转导通路在肺癌进展中的作用可能通过RhoA和TIMP-3发挥作用。Pennacchietti等^[25]^则指出低氧能促进A549等肿瘤细胞的侵袭和迁移, 进一步研究发现低氧可导致c-Met的过表达, 从而引起HGF/c-Met信号转导通路的放大, 促进肿瘤细胞包括肺癌细胞株的侵袭和迁移。而抑制c-Met的表达, 低氧诱导的肿瘤侵袭生长将被抑制。

## 4 以HGF/c-Met为靶点的抗肿瘤治疗

HGF和c-Met在肺癌的形成和演进中起着至关重要的作用, 因此, 当异常活化的HGF/Met信号通路被阻断时, 肿瘤细胞就会出现形态改变、增殖减缓、成瘤性降低、侵袭能力下降等一系列的变化, 提示HGF/c-Met是一个在肿瘤治疗中有效的分子靶点。目前, 以HGF/c-Met为靶点的肿瘤分子靶向治疗药物主要包括有拮抗剂、抗体、小分子抑制剂等, 表 1列出了HGF/c-Met通路抑制剂、抑制机制及应用情况。

**1:** HGF/c-Met通路抑制剂, 抑制机制及应用情况 HGF/c-Met pathway Inhibitors developed for lung cancer and the other solid tumors

Compound	Company	Mechanism of action	Phase/Type of tumor	Ref
Antagonists				
NK2/NK4		Inhibit HGF binding to Met	Preclinical/Solid tumors	[41, 55]
Uncleaved HGF		Inhibit HGF activation	Preclinical/Solid tumors	[56]
Decoy MET	Compugen	Inhibit MET activationr	Preclinical/NSCLC	[57]
Antibodies				
Rilotumumab	Amgen	Anti-HGF (IgG2)	Phase Ⅰ-Ⅱ/NSCLC	NCT01233687
Ficlatuzumab	AVEO	Anti-HGF (IgG1)	Phase Ⅰb-Ⅱ/NSCLC	NCT01039948
TAK-701	Millennium	Anti-HGF (IgG1)	Phase Ⅰ/Solid Tumors	NCT00831896
Onartuzumab	Genentech	Anti-Met (IgG1)	Phase Ⅲ/ NSCLC	NCT01456325
OA-5D5	Genentech	Anti-Met	Preclinical/ NSCLC	[58]
DN30	Metheresis	Anti-Met	Preclinical/ GTL16	[59]
CE-355621	Pfizer	Anti-Met	Preclinical/ U87-MG	[60]
Small-molecule c-Met inhibitors				
Tivantinib	ArQule	Selective, Met TKI (Non-ATP)	Phase Ⅲ/NSCLC	NCT01377376
AMG337	Amgen	Selective, Met TKI (ATP)	Phase Ⅰ/Solid tumors	NCT01253707
SGX523	SGX Pharma.	Selective, Met TKI (ATP)	Phase Ⅰ /Solid tumors	NCT00606879
AMG 208	Amgen	Selective, Met TKI	Phase Ⅱ/DLBCL	NCT01740792
PF-04217903	P*fi*zer	Selective, Met TKI (ATP)	Phase Ⅰ/Solid tumors	NCT00706355
EMD 1214063	EMD Serono	Selective, Met TKI	Phase Ⅰ/Solid tumors	NCT01014936
BMS777607	Bristol-Myers Squibb	Selective, Met TKI (ATP)	Phase Ⅰ-Ⅱ/Solid tumors	NCT00605618
JNJ38877605	Johnson & Johnson	Selective, Met TKI	Phase Ⅰ/Solid tumors	NCT00651365
INCB28060	Incyte& Novartis	Selective, Met TKI (ATP)	Phase Ⅰ/Advanced cancer	NCT01072266
PHA665752	Tocris Bioscience	Selective, Met TKI (ATP)	Preclinical/NSCLC	[61]
Crizotinib	Pfizer	Selective, Met TKI (ATP)	Phase Ⅲ/ALK-altered NSCLC Ph Ⅰ-Ⅱ/ solid tumors	NCT01639001
Golvatinib	Eisai	TKI of c-Met and VEGFR	Phase Ⅰ-Ⅱ/Solid tumors	NCT01433991
Cabozantinib	Exelixis	Receptor TKI (ATP)	Phase Ⅱ/NSCLC Ph Ⅲ/ solid tumors	NCT01708954 NCT01908426
Foretinib	Exelixis	Receptor TKI (ATP)	Phase Ⅰ-Ⅱ/Solid tumors	NCT01068587
MGCD265	MethylGene	TKI of Met and VEGF	Phase Ⅱ/NSCLC	NCT00975767
NCT References are available on http://clinicaltrials.gov/ct2/home; NSCLC:non-small cell lung cancer; DLBCL:diffuse large B cell lymphoma; TKI:tyrosine kinase inhibitors; VEGF:vascular endothelial growth factor; HGF:hepatocyte growth factor.				

### 4.1 生物拮抗剂

HGF的生物拮抗剂NK2、NK4及NK1都是HGF的变异体, 可与c-Met结合, 竞争性地抑制HGF和c-Met的相互作用。因其本身不能诱导c-Met的酪氨酸磷酸化, 影响HGF/c-Met系统的信号转导, 从而抑制HGF所诱导的细胞的增殖、运动和迁移等。Kishi等^[41]^采用腺病毒介导裸鼠体内高表达NK4, 发现NK4明显抑制B16F10黑素瘤和Lewis肺癌细胞的肿瘤生长及肺转移, 提示NK4是一种抑制肿瘤生长的抑制剂, 可利用NK4进行基因治疗。

### 4.2 单克隆抗体

#### 4.2.1 HGF抗体

HGF抗体能够中和HGF的活性, 阻止HGF与c-Met的结合, 代表物有AMG102、AV-299、TAK701等。AMG102也称Rilotumumab, 是Amgen公司研究的中和HGF的抗人单克隆IgG2抗体, 可阻止HGF与c-Met的结合及其介导的c-Met磷酸化和信号转导。AMG102的抗原表位可结合到HGFβ链的NH2末端, 此区域是HGF与c-Met相互作用的关键位点。免疫沉淀实验显示AMG102可有效地与成熟的有活性的HGF相结合。研究^[42]^还发现, AMG102可增强替莫唑胺(Temozolomide)和多西他赛(Docetaxel)在U87-MG细胞和动物模型中的抗肿瘤效果。比利时Van Cutsem等^[43]^在EGFR单抗基础上, 联合使用HGF抑制剂Rilotumumab用于晚期结直肠肿瘤的治疗, 确定了Rilotumumab的安全性。目前, 一项AMG102和厄洛替尼用于晚期的非小细胞肺癌患者的Ⅰ期/Ⅱ期临床研究正在进行中(Identifier:NCT01233687, http://clinicaltrials.gov/ct2/home, 下同)。

Ficlatuzumab(也称AV-299)是Aveo公司开发针对HGF的单克隆抗体, Ⅰ期临床研究显示接受Ficlatuzumab与EGFR小分子抑制剂吉非替尼或厄洛替尼相结合治疗, 患者能很好地耐受。二期临床研究中, 接受吉非替尼及组合药物(Ficlatuzumab和吉非替尼)治疗的患者组, 总响应率分别为40%和43%, 平均无进展生存期分别为4.7个月和5.6个月。接受组合药物的患者组表现出了较好的疗效和更长的无进展生存期, 虽然两组无统计学意义, Ficlatuzumab在二期临床研究中并没有取得预期的效果。目前该药临床研究还在进行中(Identifier:NCT01039948), 以期找到更好的使用途径。

#### 4.2.2 针对c-Met的单克隆抗体

以c-Met为靶点的抗体亦可阻止HGF的结合及抑制c-Met二聚体化, 代表性化合物有c-Met的单克隆抗体Onartuzumab(metMAb), 是由Genentech公司开发的人源化的抗c-Met的单克隆抗体, 可阻止HGF与c-Met的结合及下游信号通路的活化及信号转导。metMAb抑制HGF/c-Met介导的肿瘤生长, 一项Ⅰb期临床试验确定了metMAb的安全性和建议剂量。最近, 一项全球性的、随机的、双盲的Ⅱ期临床试验对比metMAb加厄洛替尼以及安慰剂加厄洛替尼在非小细胞肺癌的二、三线治疗效果^[44]^。128例患者分为两组, 分别使用MetMAb加厄洛替尼以及安慰剂加厄洛替尼治疗方案。c-Met阳性的非小细胞肺癌患者, 接受MetMAb加厄洛替尼治疗组患者的无进展生存期(progression free survival, PFS)及总生存期(overall survival, OS)有延长。目前, 该药物的Ⅲ期临床研究正在进行中(Identifier:NCT01456325)。

以c-Met为靶点的抗体药物还有Lily公司的LY2875358, 目前在非小细胞肺癌Ⅱ期临床研究正在进行中(Identifier:NCT01456325, NCT01897480)。

### 4.3 c-Met小分子抑制剂

c-Met小分子抑制剂分为非选择性和选择性的酪氨酸激酶抑制剂, 其中非选择性抑制剂主要有Crizotinib、Cabozantinib、Foretinib、Golvatinib等, 而选择性抑制剂有Tivantinib、AMG337、BMS-777607、SGX523等。

#### 4.3.1 非选择性抑制剂

##### 4.3.1.1 Crizotinib

Crizotinib(又名PF-02341066, 商品名:Xalkori)由辉瑞公司开发的针对c-Met蛋白、ALK以及RON的小分子ATP竞争性抑制剂。体外研究证实, Crizotinib抑制c-Met酪氨酸残基与下游Akt、ERK的磷酸化, 并抑制细胞的增殖和细胞粘附。Crizotinib可抑制肿瘤细胞c-Met的表达。Shaw等报告了一项新的Ⅲ期临床试验^[45]^显示:对于接受过以铂类为基础一线化疗的ALK阳性非小细胞肺癌患者, Crizotinib治疗比标准化疗更为有效。ALK阳性患者接受Crizotinib二、三线治疗后, 生存情况明显改善。这些结果提示了:Crizotinib可以作为ALK阳性非小细胞肺癌晚期患者的标准治疗方案。目前一项包含334例ALK阳性的非鳞癌患者参加的研究将对Crizotinib和培美曲塞+顺铂或培美曲塞+卡铂的疗效和安全性进行评价和比较, 该研究预期在2013年12月完成(Identifier:NCT01639001)。

##### 4.3.1.2 Cabozantinib

Cabozantinib(又名XL184), 是由Exelixis研发的广谱激酶抑制剂, 通过靶向抑制c-Met、VEGFR2及RET信号通路而发挥抗肿瘤作用, 它能够杀死肿瘤细胞, 减少转移并抑制血管生成。一项涉及330例甲状腺髓样癌患者的临床研究确定了cabozantinib的安全性和有效性。Cabozantinib组患者的PFS为11.2个月, 而安慰剂组为4个月。结果还显示, Cabozantinib组有27%的患者肿瘤体积在15个月的时间里有所缩小, 而安慰剂组肿瘤未出现体积缩小现象^[46]^。2012年11月29日, 美国FDA批准了Cabozantinib(商品名Cometriq)用于治疗转移性甲状腺髓样癌, 可见FDA对Cabozantinib在临床试验中的PFS的延长和轻微毒性反应的认可。目前, 该药联合厄洛替尼用于Ⅳ期的非小细胞肺癌患者的二线和三线治疗的二期临床试验正在进行中(Identifier:NCT01708954)。

##### 4.3.1.3 Foretinib

Foretinib(又名XL880)是Exelixis公司研发的ATP竞争性的广谱酪氨酸激酶抑制剂, 主要作用于c-Met和VEGFR, 最近, 一项包括74例乳头状肾细胞癌患者参与的Ⅱ期临床研究确定了Foretinib对肾细胞癌的疗效与安全性^[47]^, 研究人员将患者分为两组:间歇给药组, 在每14天中的第1-第5天内每日服用1次Foretinib, 每次240 mg; 每日给药组, 每日80 mg Foretinib。最后患者ORR为13.5%, 中位无进展生存期为9.3个月。该研究显示Foretinib对于晚期乳头状肾细胞癌患者的疗效, 并且其毒性可控, 对存在*c-Met*基因突变的患者具有较高的缓解率。目前该药针对非小细胞肺癌的研究处于临床Ⅰ期/Ⅱ期阶段(Identifier:NCT01068587)。

##### 4.3.1.4 Amuvatinib

Amuvatinib(又名MP-470), 为多靶点c-Kit、c-Met、PDGFα和FLT3抑制剂。MP-470在体外能有效抑制前列腺癌细胞的增殖, 并促进细胞凋亡。MP-470联合厄洛替尼能抑制LNCaP移植瘤小鼠中前列腺癌细胞增殖, 其肿瘤抑制率达到30%-65%^[48]^。一项100例患者参与的Amuvatinib与其他5种标准化疗方案联合用药的Ⅰb期临床试验显示, Amuvatinib与紫杉醇、Amuvatinib与依托泊苷合用组显示出较强的抗肿瘤活性, 同时小细胞肺癌和神经内分泌肿瘤对于Amuvatinib较敏感, 46%患者疗效达到PR^[49]^。目前, Amuvatinib治疗小细胞肺癌的研究处于临床Ⅱ期阶段(Identifier:NCT01357395)。

##### 4.3.1.5 MGCD-265

MGCD-265是多靶点的, ATP竞争性的c-Met和VEGFR1/2/3抑制剂, MGCD-265作用于c-Met过表达的MDA-MB-231、COLO205和A549移植瘤的小鼠, 可抑制肿瘤生长和c-Met信号通路。目前MGCD-265治疗晚期恶性肿瘤已经完成Ⅰ期临床试验研究。最近, MGCD-265和厄洛替尼或多西他赛(Docetaxel)联用作用于晚期恶性肿瘤或非小细胞肺癌, 正处于Ⅰ期/Ⅱ期临床试验研究阶段(Identifier:NCT00975767)。

#### 4.3.2 选择性抑制剂

##### 4.3.2.1 Tivantinib(ARQ-197)

Tivantinib是由美国ArQule公司与日本Daiichi Sankyo和Kyowa Hakko Kirin公司联合开发的一种具口服活性的高选择性c-Met抑制剂, 目前该药治疗非小细胞肺癌的研究处于临床Ⅲ期阶段, 针对肝癌、胰腺癌、胃癌等处于临床Ⅱ期阶段。不同于典型的小分子酪氨酸激酶抑制剂, Tivantinib是以非ATP竞争性方式结合于未磷酸化或未激活的受体来阻滞受体的激活及下游信号转导。体内外实验显示, Tivantinib能明显抑制具c-Met表达或过表达的人结肠腺癌HT-29、胃癌MKN-45、乳腺腺癌MDA-MB-231等细胞株的增殖及caspase依赖性凋亡, 经口服可有效抑制移植瘤小鼠的肿瘤生长^[50]^。一项由167例晚期非小细胞肺癌患者参加的双盲、随机Ⅱ期临床试验结果显示, 厄洛替尼联合Tivantinib组中位PFS为3.8个月, 厄洛替尼联合安慰剂组中位PFS为2.3个月。在*K-ras*突变患者中, 厄洛替尼与Tivantinib联用的PFS获益明显。研究^[51]^显示, Tivantinib和厄洛替尼联用耐受性良好。尽管该研究没有达到预期的效果, 但Tivantinib的疗效, 特别是在存在*K-ras*突变患者中的疗效得到了证实。

##### 4.3.2.2 AMG337

AMG337是由美国Amgen公司开发的一种口服的高选择性c-Met抑制剂。目前, 该药尚处于临床前开发, 一项AMG337治疗晚期实体瘤的的研究处于Ⅰ期临床试验阶段(Identifier:NCT01253707)。

##### 4.3.2.3 JNJ-38877605

JNJ-38877605是Johnson & Johnson公司开发的小分子ATP竞争性c-Met抑制剂, 有效抑制HGF刺激的和组成型激活的c-Met磷酸化。JNJ-38877605能明显降低EBC1、TL16、NCI-H1993和MKN45细胞的c-Met和RON磷酸化。最新研究^[52]^显示JNJ-38877605作用于GTL16细胞, 导致IL-8、GROa及可溶性尿激酶受体uPAR分泌下调, 并促进IL-6的调节分泌。目前, JNJ-38877605治疗恶性肿瘤的Ⅰ期临床研究已经完成。

##### 4.3.2.4 PF-04217903

PF-04217903是选择性的ATP竞争性c-Met抑制剂, 其选择性非常高, 对c-Met致癌突变更为敏感。PF-04217903能有效抑制c-Met驱动的生物进程, 如多种肿瘤细胞的生长、运动、侵袭和形态学变化等。PF-04217903和Sunitinib联用作用于对Sunitinib敏感的EL4和LLC肿瘤模型, PF-04217903和Sunitinib联用能明显阻断血管扩张而抑制肿瘤生长^[53]^。目前, PF-04217903治疗晚期肿瘤已经完成Ⅰ期研究阶段(Identifier:NCT00706355)。

##### 4.3.2.5 SU11274

SU11274是一种具有吲哚酮化合物的小分子酪氨酸激酶抑制剂, 作用于c-Met通路, 除了抗血管生成作用外, 还有抗细胞增殖的活性。SU11274在体外能明显抑制c-Met自身磷酸化位点的磷酸化, 从而抑制其下游信号的传导。SU11274抑制大肠癌Lovo细胞的增殖及诱导细胞周期停留在G_1_期, 并且抑制裸鼠大肠癌移植瘤的生长^[54]^。目前该药还处于临床前研究阶段, 有望成为恶性肿瘤的治疗药物之一。

综上所述, HGF/c-Met信号通路在肺癌和多种实体瘤发生和侵袭转移中发挥着重要的作用, c-Met过表达和突变与多种肿瘤包括肺癌的发病机制有关, 因此, HGF/c-Met成为非小细胞肺癌治疗中很有前途的靶标, 多种临床前及临床研究都已经阐明了HGF/c-Met信号通路抑制剂在非小细胞肺癌中的作用。根据患者c-Met扩增、突变、过度表达情况和血清HGF表达水平, 以及*EGFR*和*K-ras*突变等情况, 筛选哪些患者适于何种治疗, 确定哪些患者适于传统治疗而哪些患者进行靶向治疗, 靶向治疗时是单一用药还是联合用药。
